# Advancements in triple-negative breast cancer sub-typing, diagnosis and treatment with assistance of artificial intelligence : a focused review

**DOI:** 10.1007/s00432-024-05903-2

**Published:** 2024-08-06

**Authors:** Zahra Batool, Mohammad Amjad Kamal, Bairong Shen

**Affiliations:** 1 Center for High Altitude Medicine, West China Hospital, Sichuan University, Chengdu, 610041 China; 2 West China Tianfu Hospital, Sichuan University, Chengdu, Sichuan 610218 China; 3 King Fahd Medical Research Center, King Abdulaziz University, Jeddah, 21589 Saudi Arabia; 4 Enzymoics, Novel Global Community Educational Foundation, Hebersham, NSW 2770 Australia; 5 Joint Laboratory of Artificial Intelligence for Critical Care Medicine, Department of Critical Care Medicine and Institutes for Systems Genetics, Frontiers Science Center for Disease-related Molecular Network, West China Hospital, Sichuan University, A-10, No.17, Tianfu Avenue, Shangliu Distinct, Chengdu, 610002 China

**Keywords:** Artificial intelligence, Triple negative breast cancer, Machine learning, Deep learning, Algorithms, Treatment

## Abstract

Triple negative breast cancer (TNBC) is most aggressive type of breast cancer with multiple invasive sub-types and leading cause of women’s death worldwide. Lack of estrogen receptor (ER), progesterone receptor (PR), and human epidermal growth factor receptor 2 (HER-2) causes it to spread rapidly making its treatment challenging due to unresponsiveness towards anti-HER and endocrine therapy. Hence, needing advanced therapeutic treatments and strategies in order to get better recovery from TNBC. Artificial intelligence (AI) has been emerged by giving its high inputs in the automated diagnosis as well as treatment of several diseases, particularly TNBC. AI based TNBC molecular sub-typing, diagnosis as well as therapeutic treatment has become successful now days. Therefore, present review has reviewed recent advancements in the role and assistance of AI particularly focusing on molecular sub-typing, diagnosis as well as treatment of TNBC. Meanwhile, advantages, certain limitations and future implications of AI assistance in the TNBC diagnosis and treatment are also discussed in order to fully understand readers regarding this issue.

**Graphical Abstract:**

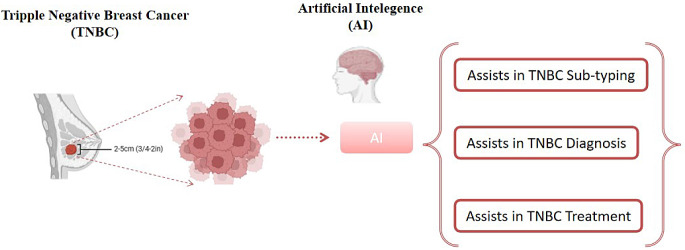

## Introduction

Artificial intelligence (AI) has emerged as a dynamic technological discipline focused on the advancement of scientific theories and methods to model and enhance human intelligence (Ali et al. 2022). As AI continues to evolve, its applications extended across diverse domains, including healthcare, communication media, and various technological fields. In the medical realm, AI has made significant strides in aiding diagnostics and treatment procedures for various diseases.

**Fig. 1:**
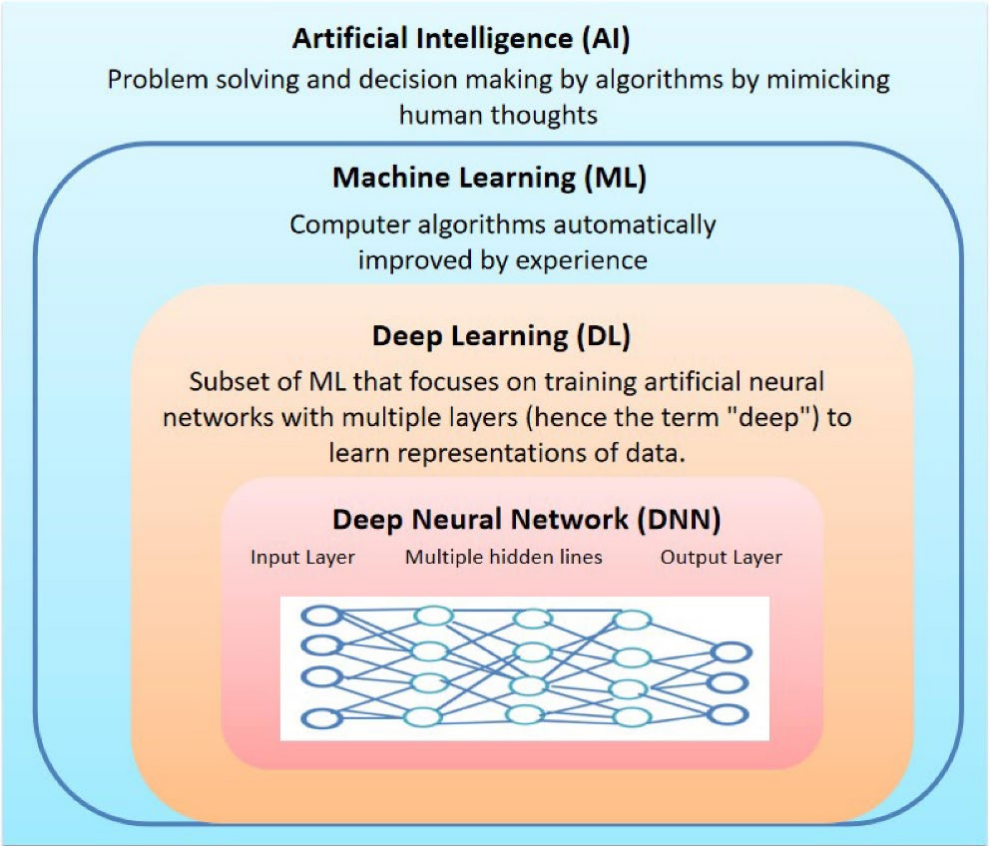
In depth classification of artificial intelligence

Presently, AI is primarily composed of two key components: machine learning (ML) and deep learning (DL) (Mendelson 2019) (Fig. 1). ML utilizes specific algorithms to learn from vast datasets, enabling the creation of decision rules, enhancing the identification of common characteristics across different data types, thereby improving diagnostic accuracy (Yan et al. 2023). On the other hand, DL, a subset of machine learning (Bluemke et al. 2020), employ computers to process large volume of data for training, validation, and testing. DL algorithms automatically extract and classify abstract features from images, contributing to the advancement of diagnostic capabilities. The implementation of the DL methods has proven to be more straightforward and precise compared to ML (Yan et al. 2023; Bluemke et al. 2020). The rise of various neural networks such as convolutional neural networks (CNNs), fully convolutional networks (FCNs), recurrent neural networks (RNNs), and generative adversarial networks (GANs) has facilitated significant advancements in computational power processing. These advancements did not only popularized algorithms, however also led to the widespread application of DL-based AI in cancer pathology (Jiang et al. 2020) holding promise in aiding radiologists. The application of AI including ML and DL, in healthcare offers numerous advantages, such as streamlined data collection and processing, as well as programming surgical robots (Ullah et al. 2020). By quantifying subtle information within images that may escape human detection, AI can serve as a valuable complement to clinical decision-making (Ali et al. 2022). Moreover, it has the capability to integrate diverse data streams into a robust diagnostic system, encompassing radiographic images and genomics data (Kumar et al. 2022). Likewise, AI has found applications in the analysis of digital pathology images for various tasks, including cancer diagnosis, prognosis, and treatment prediction (Ali et al. 2022; Mendelson 2019; Yan et al. 2023). DL and ML techniques have been leveraged for the analysis of digital pathology images, enabling the differentiation of cancer types, identification of tumor components, detection of gene mutations, assessment of grading status, and prediction of prognosis and disease recurrence (Ullah et al. 2020; Valieris et al. 2020).

Recently, AI has played a crucial role, especially in breast cancer management, through the creation of a web-based software ecosystem known as Decision Support and Information Management System for Breast Cancer (DESIREE)(Redjdal et al. 2021). DESIREE is actively involved in the individualized diagnosis as well as treatment of BC followed by complete follow up after recovery (Redjdal et al. 2021; Pelayo et al. 2021). Triple-negative breast cancer (TNBC) has been evolved as most aggressive type of BC (Vagia et al. 2020) due to its lack of estrogen receptor (ER), progesterone receptor (PR), and human epidermal growth factor receptor 2 (HER-2), resulting in un-responsiveness towards endocrine therapy or anti-HER2 treatment (Vagia et al. 2020).

Moreover, metastatic TNBC at stage IV spreads to other parts of the body, resulting in an overall survival rate ranging from 4 to 20% over a five-year time span (Zhang and Yeung 2023). Understanding the heterogeneity of metastasized TNBC within the tumor micro-environment is crucial for developing advanced treatment options. Furthermore, mutations in BRCA1/BRCA2 are commonly associated with breast cancer risk, particularly prevalent in TNBC patients across all stages, from early (stage I) to metastatic (stage IV) stage (Yu and Di 2017). Approximately 20% of TNBC patients carry these mutations, underscoring the importance of genetic screening for individuals diagnosed with TNBC (Hu et al. 2020).

In the development of chemotherapy options for metastatic TNBC, platinum-based chemotherapy has demonstrated advantages in progression-free survival (PFS) compared to non-platinum-based regimens (Pandy et al. 2019). However, its efficacy is context-dependent, particularly benefiting TNBC cases with BRCA1/BRCA2 mutations than other homologous recombination deficiencies. Consequently, there remains a critical need to advance therapeutic options that can effectively treat metastatic TNBC across all genetic mutations, not solely limited to BRCA1/BRCA2 mutations (Pandy et al. 2019).

Moreover, several FDA-approved treatments for breast cancer are increasingly being explored for the management of metastatic TNBC. These include targeted therapies such as PARP inhibitors, immune checkpoint inhibitors, antibody-drug conjugates (ADCs), and emerging novel drug agents. However, the treatment of metastatic TNBC remains challenging due to factors like clonal diversity, increased tumor mutation burden, and both germ-line and somatic mutations in genes associated with homologous recombination DNA repair. These complexities complicate the development of effective targeted therapies and novel drug agents for this heterogeneous disease (Saini et al. 2021), hence needing AI technologies to cope up with these challenges and developing authentic therapeutic treatments for metastatic TNBC by handling clinical and molecular data.

Consequently, chemotherapy, surgery, and radiotherapy remain the primary treatment modalities for TNBC from stage I to stage IV (metastatic TNBC). In recent years, advancements in computational technology led to the emergence of several AI-based approaches, offering robust analytical frameworks for handling TNBC clinical and molecular data (Ali et al. 2022). These advancements have significantly contributed in the sub-typing, diagnosis and treatment of TNBC from stage I to stage IV (metastatic TNBC). Hence, this review has deeply reviewed recent advancements of AI assistance for TNBC sub-typing, diagnosis, and treatment.

## Searching strategy

To conduct a comprehensive search of relevant studies on the role of AI assistance in the sub-typing, diagnosis, and treatment of Triple-Negative Breast Cancer (TNBC), various electronic databases and registers including CINAHL (EBSCOhost), MEDLINE (EBSCOhost), APA (PsycINFO), Cochrane Library (CENTRAL register), Google scholar, Scopus and Web of Science Core Collection were systematically searched. Assistance from an expert librarian was sought to ensure thoroughness and accuracy in the searching process. The search was limited to studies published between 2019 to early 2024 to capture the most recent and relevant literature. Studies were included if they investigated the role of AI assistance in sub-typing, diagnosing, or treating TNBC. Any studies that did not address AI assistance for TNBC were excluded from the search and were not included in the review process. Hence, ensuring that only studies directly relevant to the research were considered for inclusion, thus maintaining the quality and high relevance of the review.

## AI in TNBC sub-typing

### Problems in TNBC sub-typing

Growing evidence underscores the heterogeneity of TNBC, with numerous studies focusing on identifying TNBC sub-types through genomic analysis (Zhao et al. 2020). However, molecular sub-typing holds promise in guiding precise treatments tailored to the specific sub-types of TNBC (Tsang and Tse 2023). Such as study of Ensenyat-Mendez et al., (2021) (Ensenyat-Mendez et al. 2021) conducted research on transcriptome-based TNBC sub-types from 465 TNBC cases, and analyzed data based on their genomic, clinical as well as transcriptomic profiles. Authors classified these cases into four TNBC sub-types: basal-like immune-suppressed (BLIS), immunomodulatory (IM), luminal androgen receptor (LAR) and mesenchymal-like (MES). Their study revealed that these tumor-specific sub-types exhibited distinct genetic alterations, necessitating different treatment strategies. Meanwhile, a recent study by Hu et al., (2024) (Hu et al. 2024) has performed genomic profiling by using immunohistochemistry for TNBC sub-typing as a practical approach. However, challenges such as variability in molecular data and the lack of cost-effective systematic classification methods hinder the implementation of these advances in TNBC sub-typing.

### AI assistance in TNBC sub-typing

**Fig. 2:**
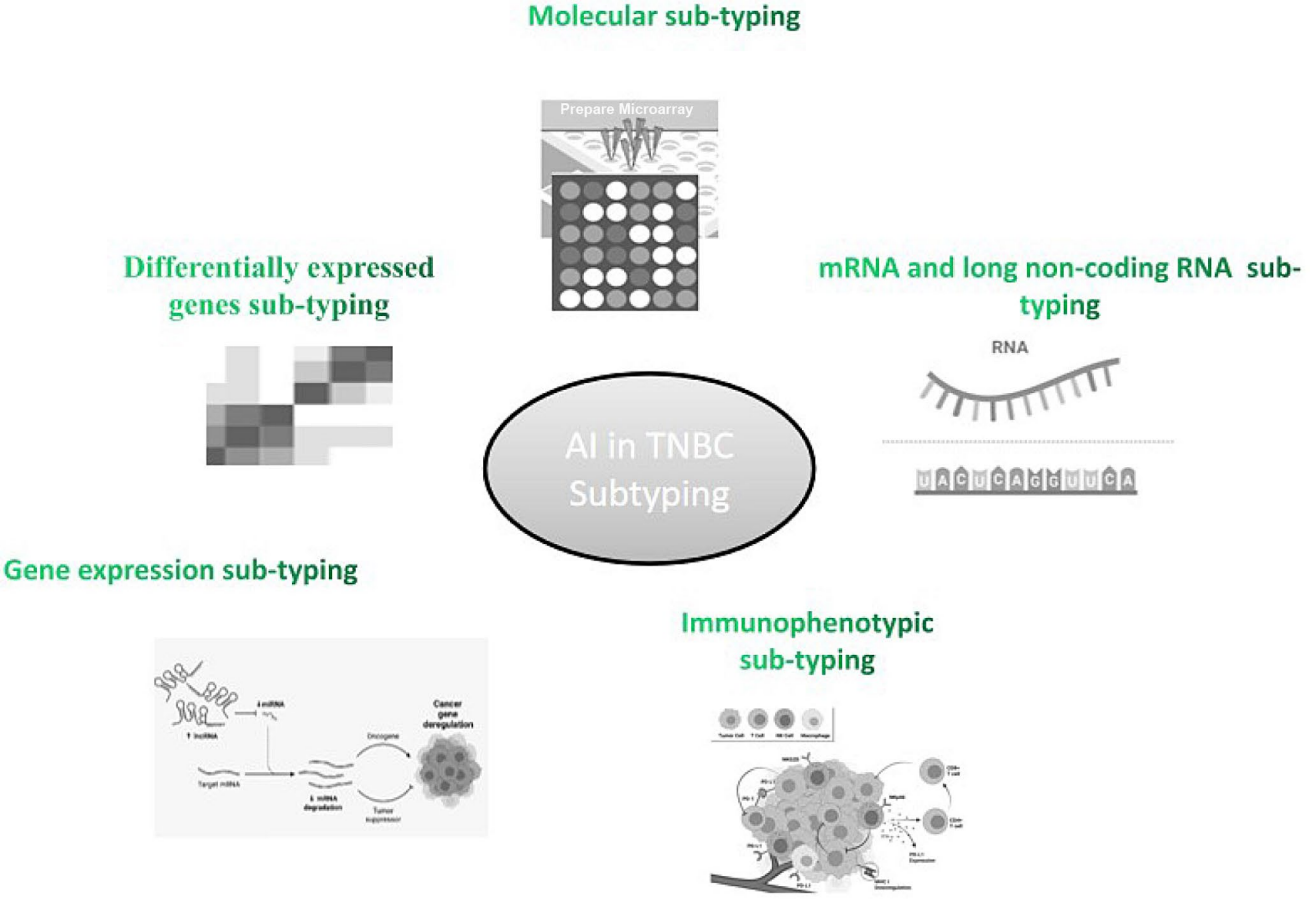
A framework for enhanced gene selection performance, gene connectivity and distinguishing and classification of TNBC isoforms

AI has tried to solve these TNBC sub-typing issues by its assistance in performing different sub-typing protocols (Fig. 2). This section will review these research studies dealing AI assistance in TNBC sub-typing (Table 1).

**Table 1:** Summary of all research studies conducted to perform TNBC sub-typing by assistance of AI

Study Type	Aim of study	Identification	Strategy	Outcomes/Algorithms	Performance	AUC	References
TNBC molecular sub-typing	To address the lack of consensus in TNBC molecular sub-typing	Rational design of TNBC sub-typing	Utilization computational tools and algorithms.	Obtained TNBC sub-typing methods and algorithms	Algorithms for TNBC sub-typing gave highest performance	0.86	(Yu et al. 2022)
TNBC sub-typing based on mRNA and long non-coding RNA	To distinguish between six isoforms of TNBC	To identify mRNA and long non-coding RNA expression data	Developed a gene selection method, DGGA	Obtained optimal subset and intra-gene correlation	TNBC isoform classification	0.91	(Liu et al. 2021)
TNBC sub-typing based on immunophenotypic profiling	To obtain stratification of TNBC patients	To identify two new immune subtypes, termed S1 and S2.	Immunophenotypic analysis and bioinformatics	Constructed machine learning model based on the Random Forest (RF) algorithm	ML model gave high performance	0.97	(Chen et al. 2021)
TNBC sub-typing based on gene expression	To classify TNBC patients	Identification between two CAF sub-types (CAF + and CAF−)	Cluster-free analysis of gene expression profiles	Constructed a robust random forest (RF) model	RF model gave high performance	0.921	(Wang et al. 2022)
TNBC sub-typing based on deferentially expressed genes (DEGs)	To develop TNBC classification models	To identify deferentially expressed genes	Utilizing DEGs as training gene set	Obtained SVM algorithm with high accuracy compared to other ML algorithms in TNBC sub-typing	SVM gave high performance	0.99 to 1.00	(Bissanum et al. 2021)
TNBC sub-typing based on absolute assignment predictor	To cluster genomic datasets of a TNBC cohort	To identify absolute assignment predictor	Employed the transformed optimal variables in three prediction models: gradient boosting (GB), random forest (RF), and extreme gradient boosting.	Obtained models for TNBC sub-typing	GB demonstrated superior performance	0.98	(Ben Azzouz et al. 2021)

Recently, Yu et al., (2022) (Yu et al. 2022) has advanced the utilization of computational tools for TNBC sub-typing and identified challenges associated with formalin-fixed, paraffin-embedded samples and effect of batch removal across microarray platforms. Authors proposed a methodology to conduct TNBC molecular sub-typing for addressing the dearth of consent in molecular sub-typing because of disparity in sampling methods, workflow choices, computational tools, and algorithms, hence facilitated perceptive design of further research on TNBC.

**Fig. 3:**
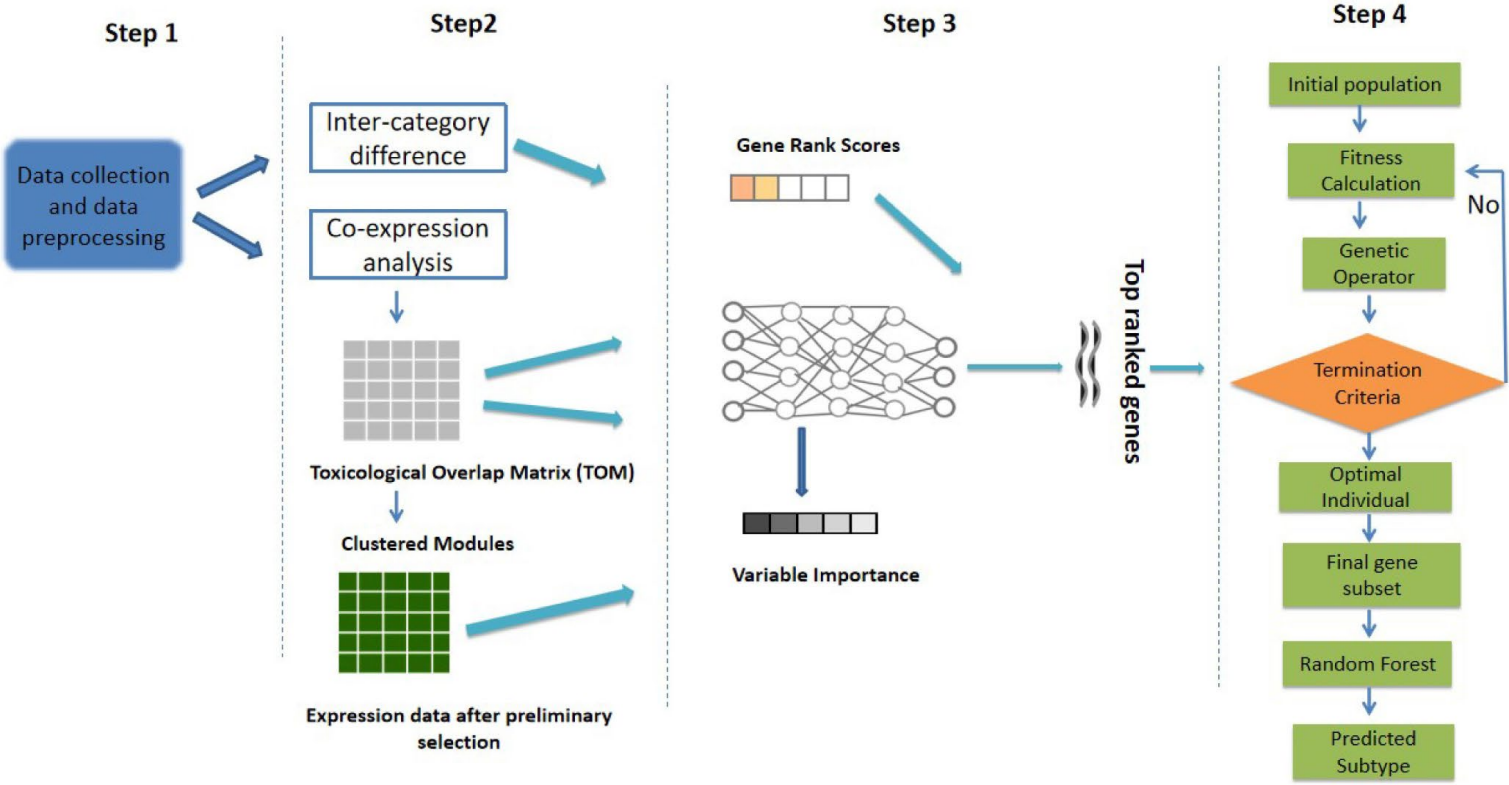
AI assistance in TNBC sub-typing while performing different protocols

Moreover, a precise and consistent classification of the six sub-types of TNBC is crucial for tailoring individualized treatments. Hence, study of Liu et al., (2022) (Liu et al. 2021) introduced a novel framework aimed at distinguishing between the six isoforms of TNBC, making it an interesting study dealing with mRNA and long non-coding RNA expression data for classification of TNBC isoforms. Notably, a gene selection method called DGGA was developed (Liu et al. 2021), incorporating correlation information among genes for effective measurement and eliminating redundant genes from consideration. Meanwhile, a gene scoring method was proposed, integrating GeneRank score with gene importance determined by a deep neural network (DNN), aiming to enhance gene selection performance. In addition to discriminating between sub-types and considering intra-gene correlation, their method incorporated a gene connectivity matrix within the DNN for sparse learning. This approach accounted for weight changes during training, providing measurements of relative gene importance. Finally, authors utilized genetic algorithms to simulate the natural evolutionary process, aiming to identify the optimal subset for TNBC isoform classification (Fig. 3). A cross-validation was further performed demonstrating accurate classification results using fewer genes. The implementation of this method can be accessed on https://github.com/RanSuLab/TNBC. Meanwhile, another study conducted by Chen et al., (2021) (Chen et al. 2021) performed immunophenotypic analysis on substantial number of TNBC samples, leading to the stratification of patients affected with TNBC pathology, in two immune sub-types, SI and S2. S1 was found being associated with relatively higher levels of immune cells, immune function scores, and exhibited better prognosis towards immunotherapy, evidenced by improved relapse-free survival (RFS) and overall survival (OS) as compared with S2. Through bio-informatics analysis, a total of 11 central genes were identified, each associated with TNBC sub-types (CD3D, STAT1, LCK, IRF4, IL2RG, IFNG, CD3G, OAS2, CD247, IRF1, and IRF2). These 11 hub genes were utilized to construct a machine learning model based on the Random Forest (RF) algorithm, evidencing strong performance with an area under the curve (AUC) of 0.76. Furthermore, this study uncovered sub-type specific TNBC molecular features, including genes, their ontologies, networks, and pathways. These findings provided valuable insights for optimally stratifying TNBC patients to better respond to immuno-therapy.

In addition to this, cancer-associated fibroblasts (CAFs) have emerged as independent prognostic factors in TNBC pathology, with elevated levels correlating with poor prognoses. This may be attributed to the dual role of CAFs, promoting tumor cell proliferation, while suppressing immune cell activity, notably CD8 + T cells, within the tumor micro-environment (TME). Therefore, Wang et al., (2022) (Wang et al. 2022) performed cluster free analysis of gene expression profiles to classify TNBC patients in two sub-types of CAF (CAF + and CAF−). Authors found association of CAF − with longer overall survival (OS) as well as immune cell infiltration as compared with CAF + sub-types. Meanwhile, their study has identified nine CAF-associated markers via bio-informatics analysis, followed by constructing robust random forest model having an AUC of 0.921. This strategy was found successful for an effective identification of CAF − sub-type patients as ideal candidates for immune checkpoint blockade therapy. Hence, promoting it as promising therapeutic approach for complementing immunotherapy as well as conventional treatments (Wang et al. 2022) .

Moreover, up til now, several gene signature datasets of TNBC sub-types have been generated by different researchers using advancements in the gene chip technology. Building upon these efforts, study by Bissanum et al., (2021) (Bissanum et al. 2021) has identified a number of deferentially expressed genes (DEGs) followed by using them as training genes set in various classification models. Their outcomes highlighted the suitability of this training gene set for developing TNBC classification models. Notably, SVM algorithm demonstrated high accuracy compared to other ML algorithms, with reported accuracy ranging from 95 to 98.8% and an AUC ranging from 0.99 to 1.00. Hence, making these findings, pivotal for personalized treatment and prognosis of TNBC. Meanwhile, another study by Jé zé quel et al., (2021) (Jé zé quel et al. 2019) has classified TNBC into three potential therapeutic sub-types: molecular apocrine response (C1), immunosuppressive response (C2), and adaptive immune response (C3). However, this study was further explored and utilized by Ben Azzouz et al., (2021) (Ben Azzouz et al. 2021) for clustering the genomic datasets of TNBC cohort, followed by employing the transformed optimal variables in three prediction models as random forest (RF), gradient boosting (GB) and extreme gradient boosting models. Meanwhile, the biological characteristics of TNBC sub-types were highlighted via analysis of up to 50 strongly correlated indices. However, based on matrices subsets, a superior performance was demonstrated by GB as compared with other models. Consequently, this prediction model has aided in classification of TNBC sub-types.

## AI in TNBC diagnosis and predicting prognosis

### Problems in TNBC diagnosis and predicting prognosis

TNBC pathology requires an early screening and diagnosis before opting for any effective treatment strategy. Magnetic resonance imaging (MRI) is highly sensitive, and can distinguish between sub-types of TNBC effectively, however it need to be conducted to determine at different stages of TNBC. Meanwhile, predicting prognosis of TNBC survival is also challenging due to involvement of multiple heterogeneous prognostic bio-markers making this prediction bit complicated.

### AI assistance in TNBC diagnosis

Recently, AI has address challenges in TNBC (stage I to stage IV (metastasis) diagnosis by developing and applying certain algorithms for improving diagnostic accuracy and efficiency. Implementing central AI algorithms into TNBC screening programs could offer a consensus approach and can significantly enhance diagnostic outcomes (Shamir et al. 2024).

This section has discussed recent advancements in leveraging AI assistance in TNBC diagnostic procedures (Table 2).

**Table 2:** Summary of all research studies conducted to perform TNBC diagnosis by assistance of AI

Study Type	Aim of study	Identification	Strategy	Outcomes/Algorithms	Performance	AUC	References
AI with multiparametric MRI for TNBC diagnosis	To evaluate molecular sub-types of TNBC	Identifying molecular sub-types of TNBC	Multi-layer perception feed-forward artificial neural network (MLPANN) with MRI	Differentiated sub-types of TNBC giving high accurate results	Differentiation of TNBC sub-types	0.86	(Leithner et al. 2020)
AI combination with radiomic features of MRI for TNBC diagnosis	To differentiate molecular sub-types of TNBC	Identifying and differentiating TNBC from non TNBC	Utilized six classification algorithms to construct models	MLP classifier algorithm	High diagnostic performance in distinguishing TNBC	0.965	(Huang et al. 2021)
AI combination with neoadjuvant systemic therapy (NAST) for TNBC diagnosis	To predict pathologic complete response (pCR)	To identify pathologic complete response (pCR) status in TNBC patients	Utilized deep learning (DL) on dynamic contrast enhancement (DCE) MRI and fusion-weighted imaging in the early stages of NAST	MRI-based DL algorithm	Differentiated TNBC patients with pCR or non-pCR at an earlier stage during NAST	0.86	(Zhou et al. 2023)
AI combination with [18-F]fluorodeoxyglucose (FDG) positron emission tomography (PET)/ magnetic resonance imaging (MRI)(18 F-FDG PET/MRI)	To distinguish molecular sub-types of TNBC from BC	To identify TNBC sub-types	Machine learning (ML)-based radiomics models applied to [18-F]fluorodeoxyglucose (FDG) positron emission tomography (PET)/ magnetic resonance imaging (MRI)(18 F-FDG PET/MRI)	ML-based radiomics model application	Distinguished TNBC lesions from other BC molecular sub-types	0.887	(Romeo et al. 2022)
AI combination with mammography system	To develop automated mammography diagnostic system with convolutional neural network (CNN)	To identity high density breasts	Lesion detection, lesion registration, and malignancy prediction modules were applied	Mammography-aided diagnostic system	Diagnostic performance over conventional imaging examinations	0.86	(Li et al. 2021)
AI combination with TNBC mammography system	To develop a decision tree model	To identify TNBC by mammography	ML was applied to build models for mammography	Decision tree model	Distinguishing TNBC from other sub-types	0.91	(Ma et al. 2022)
AI combination with ultrasound for TNBC diagnosis	To enhance capability of ultrasound images by ML	To identify TNBC	ML applied for giving high grey scale (GS) and color Doppler (CD)	Standard ultrasound images	Standard vision and improved diagnostic accuracy	0.88	(Wu et al. 2019)
AI combination with ultrasound for TNBC diagnosis	To enhance capability of ultrasound images by ML	To identify TNBC	Convolutional Neural Network (CNN) based on the VGG architecture	Deep learning system TNBC images diagnosis	improved diagnostic accuracy	0.86	(Boulenger et al. 2023)
AI combination with tumour micro-environment (sTME) to identify prognosis of TNBC	AI-based spatial tumour micro-environment (sTME)	Identifying prognostic values for diagnosing TNBC	Employed deep learning (DL) based algorithm	Segmenting tissue regions into stroma, tumor and lymphocytes for computing the quantitative features, concerning their spatial relationship.	Obtained prognostic values with digital features	0.92	(Albusayli et al. 2023)
AI-combination with diagnostic biomarkers to identify prognosis of TNBC	To develop novel 25 ML algorithms-based immune infiltrating cell (IIC) associated signature of TNBC (MLIIC)	To determine the IIC-RNA expression	Employed Boruta, LassoLR, SVM, Xgboost, RF, Pamr, Ranger, RSF, Rpart, CoxPH, CoxBoost, GlmBoost, GBM, SurvReg and CTree	MLIIC signature significantly correlated with survival status validated by four independent TNBC cohorts.	Obtained high prognostic performance	0.91	(Li et al. 2023)
AI-based analysis of tertiary lymphoid structures (TLSs) and tumor budding (TB) in TNBC patients	To evaluate the relationship between TLS/TB ratios	Identifying clinical outcomes of patients with TNBC	Established a nonogram model	Positive correlation between TLS/TB index and an overall survival (OS) and relapse-free survival (RFS) of patients with TNBC	Best performance for predicting the OS and RFS of TNBC patients.	0.96	(Hou et al. 2024)

The combination of AI with multiparametric MRI radiomics has shown its promise in accurately and non-invasively distinguishing metastatic TNBC from other sub-types of breast cancer while cancer diagnostic process (Leithner et al. 2020). Furthermore, integrating AI with spectroscopic techniques, like Raman spectroscopy (RS), during biopsy of breast cancer tissues has significantly improved the accuracy of RS for TNBC prediction. Through this approach, accuracy rates of up to 96.7% have been achieved, particularly through the linear difference analysis method. In a study by Leithner et al., (2020) (Leithner et al. 2020), a synergistic performance of AI along with radiomics using MRI was assessed to evaluate the molecular sub-types of TNBC at different stages of cancer (stage I to stage IV (metastasis). The results indicated that when employing a multi-layer perception feed-forward artificial neural network (MLPANN), the overall median area under the ROC curve (AUC) for differentiating sub-types of TNBC was 0.86, ranging from 0.77 to 0.92. In addition to this, luminal A and TNBC classification has provided median AUC of 0.80 ranging from 0.75 to 0.83. Another study by Huang et al., (2021) (Huang et al. 2021) revealed that MRI derived radiomic features can predict TNBC sub-types at all stages (stage I to stage IV (metastasis). Totally, six classification algorithms were utilized for models construction, with MLP classifier showcasing precise diagnostic performance in distinguishing TNBC from non-TNBC on test datasets. The MLP classifier achieved an impressive AUC of 0.965 and an accuracy of 92.6%. Moreover, neoadjuvant systemic therapy (NAST) followed by surgery has been proven as a standard for TNBC diagnosis, providing pathologic complete response (pCR) to 50–60% TNBC patients. Meanwhile, Zhou et al., (2023) (Zhou et al. 2023) has explored the efficacy of deep learning (DL) on dynamic contrast enhancement (DCE) MRI and fusion-weighted imaging in the early stages of NAST to predict pCR status in TNBC patients. Using images from 130 TNBC patients during the development phase, the DL model achieved impressive results with an AUC of 0.97 ± 0.04 during training and 0.82 ± 0.10 during validation. When tested on an independent group of 32 patients, the model exhibited an AUC of 0.86 ± 0.03. In a subsequent prospective, blinded test group of 48 patients, the model maintained a high AUC of 0.83 ± 0.02. These findings suggested the potential of multiparametric MRI-based DL to differentiate TNBC patients with pCR or non-pCR in the breast at an earlier stage during NAST. Another study by Romeo et al., (2022) (Romeo et al. 2022) has investigated whether machine learning (ML)-based radiomics models applied to [18-F]fluorodeoxyglucose (FDG) positron emission tomography (PET)/ magnetic resonance imaging (MRI)(18 F-FDG PET/MRI) are effective for distinguishing molecular sub-types of breast cancer (BC), particularly in differentiating between triple-negative (TN) and other molecular sub-types of BC. The study involved constructing eight radiomics models based on various combinations of quantitative parameters and/or radiomics features. The model combining the first-order neighborhood grayscale-dependent matrix extracted from ADC and PET images, along with radiomics features based on large and small matrices, demonstrated the best performance (AUC 0.887, accuracy 82.8%, sensitivity 79.7%, specificity 86%). In conclusion, the application of ML-based radiomics models to 18 F-FDG PET/MRI effectively distinguished TNBC lesions from other BC molecular sub-types with high precision and non-invasively. Looking ahead, the possibility of conducting a “virtual biopsy” using radiomics features hold promise for future applications.

Furthermore, sensitivity of BC screening is influenced by mammographic breast density, as women with high density breast have been found at high risk of developing BC. This is attributed to the decreased sensitivity of high-density breasts to imaging examinations, making it challenging to detect BC pathologies in the early stages of BC (Porembka et al. 2020). However, recent advancements have addressed this diagnostic challenge by developing mammography-aided diagnostic system comprising lesion detection, lesion registration as well as malignancy prediction modules as three main components, powered by convolutional neural networks (CNNs). The system possess excellent framework and versatile CNN output, with the system’s first module capable of performing three different tasks to precisely detect lesions. Moreover, an automated mammography diagnostic system offers significant improvements in the detection and diagnostic performance over conventional imaging examinations, even in cases of extremely dense breasts, with 75% capability to detect TNBC lesions (Li et al. 2021). Meanwhile, study of Ma et al., (2022) (Ma et al. 2022) conducted research based on clinical and imaging signs for differentiating TNBC sub-types by developing ML models. Among five developed models, best performance was achieved by decision tree model for distinguishing TNBC from other BC sub-types, and provided 0.91 value of AUC, 0.947 of precision and 0.941 of specificity. Interestingly, decision tree model identified only two features—blurred margins and mammography classification—as more important for distinguishing TNBC from other sub-types. Consequently, the application of the decision tree model can facilitate better identification of the molecular sub-types of BC patients during BC diagnosis.

Regarding ultrasound diagnostics performance, it’s crucial to accurately differentiate between TNBC and non-TNBC subtypes. Machine learning (ML) techniques, such as logistic regression and classification, have facilitated this capability by analyzing ultrasound images, including greyscale (GS) and color Doppler (CD) images. Study by Wu et al., (2019) (Wu et al. 2019) revealed statistically significant greyscale (GS) and color Doppler (CD) features, with an AUC of 0.85 and 0.65, respectively. However, an increase in the AUC values (0.88) was observed while using both GS and CD together, hence denoting higher sensitivity of 86.96% and specificity of 82.91%. This combined approach enhanced the diagnostic performance for TNBC assessing images by standard vision and improved diagnostic accuracy (Wu et al. 2019). Moreover, Boulenger et al., (2023) (Boulenger et al. 2023) developed a deep learning system to automatically identify TNBC from ultrasound images only by collecting ultrasound images and clinical information. Molecular sub-types were determined based on immunohistochemistry (IHC) results. TNBC was predicted using a convolutional neural network (CNN) based on the VGG architecture. Model visualization was conducted using t-SNE analysis and significance plots. Among the 145 patients, TNBC was detected in 16 cases (11.03%). The training set comprised 115 patients (80%), while the validation and test sets consisted of 15 patients each (10%). The deep learning system exhibited strong efficiency with 0.86 of AUC, 85% accuracy, 86% specificity and sensitivity as well as 0.74 F1 score. Furthermore, the internal characterization features learned by the model demonstrated significant differences between molecular sub-type groups. Hence, deep learning system holds promise in automatically and accurately predicting TNBC prior to surgery, thereby aiding in more precise and comprehensive management strategies.

### AI assistance in predicting TNBC Prognosis

In the pathology of TNBC, there is a critical need for diagnostic biomarkers that can predict prognosis and guide immunotherapy. AI has emerged as a valuable tool in this domain, particularly in predicting the prognosis of TNBC patients. In a recent study by Li et al. (2023) (Li et al. 2023), a novel approach using 25 machine learning (ML) algorithms was employed to develop an immune infiltrating cell (IIC)-associated signature specific to TNBC (MLIIC). This signature was constructed using multiple transcriptome datasets from purified immune cells, TNBC cell lines, and clinical TNBC samples. The authors utilized the TSI index to identify IIC-RNA, which showed up-regulation in immune cells and down-regulation in TNBC cells.Various ML algorithms including Boruta, LassoLR, SVM, Xgboost, RF, and Pamr were further employed to optimize the selection of IIC-RNAs. Additionally, algorithms such as Ranger, RSF, Rpart, CoxPH, CoxBoost, GlmBoost, GBM, SurvReg, and CTree were used to establish a robust MLIIC signature. Hence, this study concluded that the MLIIC signature strongly correlated with survival outcomes, validated across four independent TNBC cohorts. Furthermore, the MLIIC signature score has demonstrated significant prognostic value through immunofluorescent staining of tissue arrays from TNBC patients (Li et al. 2023).

Meanwhile, TNBC presents challenging prognostic variables during diagnosis and risk stratification. Therefore, a study by Albusayli et al. (2023) (Albusayli et al. 2023) has investigated digital, AI-based spatial tumor micro-environment (sTME) features and their prognostic implications in TNBC diagnosis. Initially, the authors conducted tissue classification of TNBC cases, followed by employing a deep learning (DL) algorithm to segment tissue regions into stroma, tumor, and lymphocytes to compute quantitative features based on their spatial relationships. They further explored the prognostic values of these digital features using survival analysis with Cox proportional hazard models, validated through cross-validation on two independent international multi-centric TNBC cohorts. This study identified the digital stromal tumor-infiltrating lymphocytes (Digi-sTILs) score and digital tumor-associated stroma (Digi-TAS) score as strong prognostic indicators for disease-specific survival. The Digi-sTILs score yielded a C-index of 0.65 (*p* = 0.0189), while the Digi-TAS score yielded a C-index of 0.60 (*p* = 0.0437) on the TCGA cohort, validating their utility. Thus, the authors concluded that these digital features hold promise for diagnosing and stratifying risk in TNBC patients (Albusayli et al. 2023).

Subsequently, given the highly heterogeneous nature of TNBC, studies indicate significant correlations between tertiary lymphoid structures (TLSs), tumor budding (TB), and clinical outcomes in TNBC patients. However, no integrated TLS-TB profile has been established to predict patient survival. Thus, Hou et al. (2024) (Hou et al. 2024) investigated the relationship between TLS/TB ratios and clinical outcomes in TNBC patients using AI-based analysis.The authors assessed TLSs and TB and conducted AI-based analysis. They characterized various cellular subtypes within TLSs using multiplex immunofluorescence and developed a nomogram model. Their anti-tumor model revealed a positive correlation between the TLS/TB index and overall survival (OS) and relapse-free survival (RFS) in TNBC patients. Interestingly, higher percentages of CD-8 positive cells, CD45RO-positive cells, or CD20-positive cells within TLSs were associated with improved OS and RFS.The developed model demonstrated superior predictive performance compared to the classical tumor-lymph node-metastasis (TNM) staging system for OS and RFS in TNBC patients. Hence, this novel strategy utilized AI-based analysis and machine learning workflows, establishing TLS/TB as independent prognostic factors for TNBC prognosis.

In conclusion, recent advancements in AI-driven methodologies have significantly enhanced our understanding and prognostic capabilities in TNBC pathology. Studies by Li et al., (Li et al. 2023) Albusayli et al., (Albusayli et al. 2023) and Hou et al. (Hou et al. 2024) have collectively demonstrated the effectiveness of AI in developing novel biomarkers and predictive models tailored to TNBC’s complex pathology. Li et al. (Li et al. 2023) showcased the utility of a machine learning-based immune infiltrating cell signature (MLIIC) for predicting survival outcomes, validated across multiple cohorts and reinforced by immunofluorescent staining studies. Meanwhile, Albusayli et al. (Albusayli et al. 2023) introduced digital spatial tumor micro-environment features, such as Digi-sTILs and Digi-TAS scores, as robust indicators for disease-specific survival, marking a significant leap in precision medicine for TNBC. Hou et al.‘s (Hou et al. 2024) exploration of TLS/TB ratios further underscored the predictive power of AI-driven analysis, highlighting the potential of these integrated approaches to refine risk stratification and improve patient outcomes in TNBC. These studies collectively illustrated the transformative impact of AI technologies in advancing personalized treatment strategies and prognostic assessments in TNBC, offering promising avenues for future clinical applications and research directions.

## AI in TNBC treatment

### Problems in TNBC treatment

TNBC treatment from stage I to stage IV (metastasis) has always faced several challenges due to un-responsive molecular sub-types of TNBC towards treatments. Due to lack of estrogen receptor (ER), progesterone receptor (PR), and human epidermal growth factor receptor 2 (HER-2), several therapeutic treatments remains cannot fit for TNBC treatment. Hence, predicting treatment responses and identification of novel therapeutic targets are highly challenging in this regard.

### AI assistance in TNBC treatment

AI has shown its potential to revolutionize the personalized treatment of TNBC (stage I to stage IV) by leveraging various molecular and genetic data to explore potential therapeutic targets (Gadag et al. 2020). Additionally, AI can aid in predicting treatment responses for TNBC and identifying novel therapeutic targets (Tsou et al. 2020). Furthermore, AI has become instrumental in enhancing nano-carriers by facilitating the delivery of essential components such as ligands, drugs, and tracking probes to target TNBC cellular sites. Additionally, when combined with targeted drug delivery systems and multifunctional molecules, AI has the potential to revolutionize the paradigm of nano-therapeutics for TNBC treatment (Thakur and Kutty 2019). Moreover, AI has demonstrated its effectiveness in identifying selective therapeutic targets for TNBC treatment through machine learning (ML) algorithms. This section has reviewed AI assistance in TNBC treatment (Table 3).

**Table 3:** Summary of all research studies conducted to perform TNBC treatment by assistance of AI

Study Type	Aim of study	Identification	Strategy	Outcomes/Algorithms	Performance	AUC	References
AI combination with TNBC treatment targets	To develop ML algorithm	To identify TNBC treatment targets	idTRAX development for the identification treatment targets in TNBC cell lines	ML algorithm (idTRAX )	Identified therapeutic targets for TNBC	0.86	(Gautam et al. 2019)
AI combination with TNBC treatment strategies by employing potential genes	To develop ML algorithm	To identify potential genes for TNBC treatment	ML algorithm development to investigate up-regulation of down-regulation of specific genes in TNBC treatment	ML algorithm	MFGE8 and TBC1D9 were identified for TNBC treatment	0.91	(Kothari et al. 2020)
AI combination with TNBC treatment targets as novel biomarkers	Using algorithms for novel biomarkers	To identify key genes for identification of novel biomarkers in TNBC treatment	LASSO, RF, and SVM-RFE usage	Novel biomarker	TENM2, OTOG, LEPR, and HLF were found for TNBC treatment	0.87	(Guan et al. 2023)
AI-combination with neoadjuvant chemotherapy (NAC)	To develop AI-based algorithms	To identify neoadjuvant chemotherapy (NAC) outcomes in TNBC patients.	Employed AI-algorithms using automatic feature extraction on H&E and multiplex IHC images	ML models	Accurately predicted the NAC response in TNBC patients	0.7674	(Huang et al. 2023)
AI combination with TNBC treatment specifically NAST	To predict treatment response	Employing breast hollow needle biopsies undergoing NACT for TNBC.	Automate tumor detection and nuclear segmentation process	Deep convolutional neural network (DCNN)	Pathological complete response (pCR) group exhibited fewer multifocal/multicentric tumors, and higher nuclear intensity compared to the pathological partial response (pPR) group	0.86	(Dodington et al. 2021)
AI combination with TNBC treatment without NAST	To establish a metabolite panel	TNBC treatment of patients not responding to NAST	Deep learning model (DLM) usage	Metabolite panel development	Predictive efficiency of RCB-II/III was enhanced	0.97	(Irajizad et al. 2022)
AI combination with TNBC treatment by drug response	To develop TNBC treatment response prediction models and algorithms	Computational analysis of TNBC drug response	Applying chemical perturbation gene signatures and employing cross-validation techniques	Models and algorithms	TNBC treatment response prediction	0.86	(Kim et al. 2020)

In a study by Gautam et al., (2019) (Gautam et al. 2019), a ML algorithm, idTRAX was developed for the identification of treatment targets in TNBC cell lines. The results obtained by the authors clearly indicated that inhibiting AKT selectively killed CAL148 and MFM-223 cells, while inhibition of FGFR2 targeted only MFM-223 cells. This ML algorithm has shown promising capabilities in specifically identifying therapeutic targets for TNBC, thereby facilitating drug development and personalized therapy (Li et al. 2021). In another study conducted by Kothari et al., (2020) (Kothari et al. 2020), a machine learning (ML) algorithm was employed to identify two potential genes: MFGE8 and TBC1D9. The study revealed that MFGE8 was over-expressed, whereas TBC1D9 was under-expressed. Interestingly, over-expression of TBC1D9 was associated with a favorable prognosis, playing a key role in maintaining cell integrity. On the other hand, over-expression of MFGE8 was linked to a poor prognosis, primarily contributing to the tumor survival process (Kothari et al. 2020). Therefore, targeting MFGE8 with MFGE8-specific inhibitors or its effector molecules may lead towards its down-regulation. Conversely, the up-regulation of TBC1D9 presented a notable aspect in TNBC pathology, addressed by specifically targeting its regulators through vaccination, gene therapy, or by blocking active pathways involved in its down-regulation (Kothari et al. 2020). Meanwhile, Guan et al., (2023) (Guan et al. 2023) conducted a comprehensive study aimed at identifying key genes linked to TNBC pathology. Authors utilized experimental data from the Cancer Genome Atlas Database (TCGA) and employed univariate COX regression analysis in conjunction with three algorithms for survival analysis: LASSO, RF, and SVM-RFE. Subsequently, they employed multivariate COX regression analysis to construct a risk prognosis model specifically tailored to TNBC treatment. Through their investigation, four key genes closely associated with TNBC prognosis emerged as TENM2, OTOG, LEPR, and HLF. Notably, OTOG was found as a novel biomarker in this context. Survival analysis underscored the significant impact of these four key genes on Overall Survival (OS) among TNBC patients (*P* < 0.05). Findings of their experiments suggested that these four key genes hold promise as avenues for targeted therapy in TNBC patients, potentially leading to improved prognosis and enhanced survival rates after TNBC treatment.

Furthermore, in the context of TNBC pathology, assessing clinical outcomes through pre-treatment histologic images remains challenging due to limited understanding of the tumor micro-environment. Addressing this gap, Huang et al. (2023) (Huang et al. 2023) introduced IMPRESS (IMage-based Pathological REgistration and Segmentation Statistics), an automated and accurate pipeline for whole slide image (WSI) feature extraction. Utilizing H&E and multiplex IHC images (PD-L1, CD8+, CD163+), the study investigated whether AI-based algorithms could predict neoadjuvant chemotherapy (NAC) outcomes in TNBC patients. The extracted features, encompassing tumor immune micro-environment and clinical data, were employed to train machine learning models, achieving an area under the curve (AUC) of 0.7674. This approach demonstrated promising potential for leveraging AI in predicting pre-treatment responses in TNBC, facilitating more informed treatment decisions and personalized patient care (Huang et al. 2023).

Moreover, AI assistance has emerged as a promising approach to predict TNBC sensitivity towards chemotherapeutic drugs, leveraging advanced ML algorithms (Guan et al. 2023). A study by Dodington et al., (2021) (Dodington et al. 2021) has highlighted the utilization of an AI platform to predict treatment response in breast hollow needle biopsies undergoing NACT for TNBC. Several convolutional neural networks (CNN) were employed for automating tumour detection as well as nuclear segmentation process. Relatively, fewer multifocal/multicentric tumors, higher nuclear intensity as well as lower grey-level co-occurrence matrix (GLCM-COR) were exhibited by pCR group as compared with pathological partial response (gPR) group. Consequently, leveraging AI to analyze the tumor cell nuclear features guarantees to construct a predictive model for TNBC NACT response. Meanwhile, AI assisted in synthesizing the metabolic panel profiles to identify and treat patients not responding towards NACT. In particular, TNBC (stage I to Stage IV) patients achieving pCR/residual cancer burden-0 (RCB-0) or minimal residual burden (RCB-1) and other patients having moderate to extensive tumor burden (RCB-II/III) after NACT possessed high pre-treatment acetylated polyamine levels in their plasma. This metabolite panel was further established by Irajizad et al., (2022) (Irajizad et al. 2022) with two polyamines and nine additional metabolites by using deep learning model (DLM) for enhancing predictive efficacy of RCB-II/III. This model has shown superior predictive efficiency and achieved an AUC of 0.97 with 95% specificity and 85% sensitivity to identify RCB-II/III. This exceptional performance has practical implications in clinical settings, enabling the identification of TNBC patients unresponsive towards NACT.

Furthermore, Kim et al., (2020) (Kim et al. 2020) has conducted a computational analysis of metastatic TNBC drug response scores after application of chemical perturbation gene signatures as well as cross-validation techniques followed by developing TNBC treatment response prediction models and algorithms. These developed models and algorithms were further found expediting AI integration into clinical settings for TNBC treatment (Tsopra et al. 2021).

## Advantages, limitations and future implications of AI in TNBC

### Advantages

AI, particularly ML and DL techniques, are employed in the TNBC sub-typing, diagnosis as well as treatment. These AI technologies, offer new theoretical insights in the clinical settings to address TNBC as a medical challenge featured by early metastasis, relatively poor differentiation and high recurrence rates (Bai et al. 2021). For instance, the integration of AI systems into mammography has shown significant promise in enhancing the detection rate of TNBC (Porembka et al. 2020; Li et al. 2021). Beyond detection, AI-enabled mammography also facilitated to identify potential therapeutic targets for TNBC (Li et al. 2021). Moreover, AI has given its advantages in accurately and non-invasively distinguishing TNBC from other sub-types of breast cancer by combining with multiparametric MRI radiomics and has shown its promise in accurately and non-invasively distinguishing TNBC from other sub-types of breast cancer at different stages of cancer (Leithner et al. 2020; Huang et al. 2021). Furthermore, the rapid advancement of AI subfields like ML and DL is poised to revolutionize model construction by leveraging various molecular data types and digital whole slide imaging (Wang et al. 2023; Al-Thelaya et al. 2023). This development heralds a crucial tool for integrating diverse molecular data from various existing TNBC sub-type systems (Ma et al. 2022; Boulenger et al. 2023). Ultimately, it is anticipated to yield a standardized hierarchical system that can effectively guide the treatment of TNBC patients (Dodington et al. 2021; Kim et al. 2020).

### Limitations of AI

Several unresolved challenges are hindering AI applications in the clinical diagnosis and treatment of TNBC:

#### Limited impact in drug development

While AI has shown promise in screening and aiding diagnosis of TNBC, its application in drug development remains relatively weaker. Further research and innovation are needed to effectively leverage AI in this area.

#### Lack of systematic data collection and management

A significant portion of TNBC clinical data lacks systematic collation and management. Challenges include difficulty in data acquisition, lack of uniform data standards, and controversies surrounding data theory. Addressing these issues is crucial for advancing AI applications in TNBC.

#### High development cost and low adoption rates

The development cost of AI systems is often high, and there is a shortage of professional operators. Additionally, the adoption rate of AI in clinical settings remains low, partly due to these cost and expertise constraints.

#### Poor stability of AI models

Modern AI models face challenges related to poor stability, which can undermine their reliability and effectiveness in real-world clinical settings. Moreover, improving the stability of AI models is essential for enhancing their utility in TNBC diagnosis and treatment.

Meanwhile, Flaws in data integrity, the management of large datasets, limitations in effectively translating data into actionable knowledge, ethical considerations surrounding AI usage in healthcare, and regulatory approval processes collectively pose significant barriers to the clinical adoption of AI. Addressing these challenges requires interdisciplinary collaboration, robust regulatory frameworks, and ongoing research efforts aimed at enhancing the reliability and efficacy of AI technologies in healthcare settings.

### Future implication of AI

Efforts to develop methods for interpreting and explaining AI decision models are ongoing, aiming to enhance transparency and facilitating the integration of DL into clinical practice. Certainly, delving deeper in the workings of DL algorithms to understand how they analyze data and make decisions is crucial for enhancing the interpretability and stability of AI models (Bhinder et al. 2021; Perez-Lopez et al. 2024). By gaining insights into these processes, we can improve the transparency and trustworthiness of AI systems, thus overcoming barriers to their clinical translation.

Meanwhile, in order to fully leverage the potential of ML in TNBC research, it’s crucial to store well-annotated medical data in large databases. The advent of technologies such as multiplex imaging, single-cell sequencing and spatial transcriptomics can significantly enhance the quantity and quality of TNBC data tags. These advancements can enable researchers to capture detailed molecular and cellular information, offering deeper insights into TNBC biology, progression, and treatment response. By harnessing these comprehensive datasets, ML algorithms can be trained more effectively to uncover hidden patterns, identify novel biomarkers, and predict patient outcomes with greater accuracy. Ultimately, this integration of advanced technologies and robust data repositories holds immense promise for advancing TNBC research and improving patient care. The continuous advancement of AI opens up new possibilities for TNBC research, particularly through the utilization of multimodal learning to integrate medical images and holographic data. This approach holds great promise in providing comprehensive insights into TNBC, as it combines information from different sources to enhance understanding and decision-making. As algorithms improve, big data accumulates, computing power increases, and interpretability bottlenecks are addressed, the widespread clinical application of artificial intelligence in TNBC will becomes increasingly feasible. These advancements pave the way for more accurate diagnosis, personalized treatment strategies, and improved patient outcomes in TNBC management. By harnessing the power of AI, researchers and clinicians can unlock new avenues for discovery and innovation in TNBC research and clinical practice.

## Data Availability

No datasets were generated or analysed during the current study.

## Abbreviations

TNBC: Triple negative breast cancer
AI: Artificial intelligence
ER: Estrogen receptor
PR: Progesterone receptor
HER-2: Human epidermal growth factor receptor 2
ML: Machine learning
DL: Deep learning
CNNs: Convolutional neural networks
FCNs: Fully convolutional networks
RNNs: Recurrent neural networks
GANs: Generative adversarial networks
BLIS: Basal-like immune-suppressed
IM: Immunomodulatory
LAR: Luminal androgen receptor
MES: Mesenchymal-like
RFS: Relapse-free survival
OS: Overall survival
AUC: Area under the curve
CAFs: Cancer-associated fibroblasts
TME: Tumor microenvironment
DEGs: Deferentially expressed genes
RF: Random forest
GB: Gradient boosting
MRI: Magnetic resonance imaging
RS: Raman spectroscopy
MLPANN: Multi-layer perception feed-forward artificial neural network
pCR: Pathologic complete response
DCE: Dynamic contrast enhancement
FDG: [18-F] fluorodeoxyglucose
PET: Positron emission tomography
MRI(18F-FDG PET/MRI): Magnetic resonance imaging
GS: Greyscale
CD: Color Doppler
IHC: Immunohistochemistry
GLCM-COR: Lower grey-level co-occurrence matrix
gPR: Pathological partial response
RCB-0: Residual cancer burden-0

## Declarations

### Generative artificial intelligence statement

During the preparation of this work the authors used Chatgtp3.5 in order to improve the English language and grammatical mistakes in the written article. After using this service, the authors reviewed and edited the content as needed and take full responsibility for the content of the publication.

## References

[CR54] Al-Thelaya K, Gilal NU, Alzubaidi M, Majeed F, Agus M, Schneider J, Househ M (2023) Applications of discriminative and deep learning feature extraction methods for whole slide image analysis: a survey. J Pathol Inf 14:100335 10.1016/j.jpi.2023.100335 PMC10622844 37928897

[CR39] Albusayli R, Graham JD, Pathmanathan N, Shaban M, Raza SEA, Minhas F, Armes JE (2023) Rajpoot, N. Artificial intelligence-based digital scores of stromal tumour-infiltrating lymphocytes and tumour-associated stroma predict disease-specific survival in triple-negative breast cancer. J Pathol 260:32–42 36705810 10.1002/path.6061

[CR1] Ali R, Balamurali M, Varamini PD (2022) Learning-based Artificial Intelligence to investigate targeted nanoparticles’ Uptake in TNBC cells. Int J Mol Sci 23:16070 36555718 10.3390/ijms232416070 PMC9785476

[CR52] Bai X, Ni J, Beretov J, Graham P, Li Y (2021) Triple-negative breast cancer therapeutic resistance: where is the Achilles’ heel? Cancer Lett 497:100–111 33069769 10.1016/j.canlet.2020.10.016

[CR27] Ben Azzouz F, Michel B, Lasla H, Gouraud W, François AF, Girka F et al (2021) Development of an absolute assignment predictor for triple-negative breast cancer subtyping using machine learning approaches. Comput Biol Med 129:104171 33316552 10.1016/j.compbiomed.2020.104171

[CR55] Bhinder B, Gilvary C, Madhukar NS, Elemento O (2021) Artificial intelligence in cancer research and precision medicine. Cancer Discov 11:900–915 33811123 10.1158/2159-8290.CD-21-0090 PMC8034385

[CR25] Bissanum R, Chaichulee S, Kamolphiwong R, Navakanitworakul R, Kanokwiroon K (2021) Molecular classification models for triple negative breast cancer subtype using machine learning. J Pers Med 11:881 34575658 10.3390/jpm11090881 PMC8472680

[CR4] Bluemke DA, Moy L, Bredella MA (2020) Assessing radiology research on artifcial intelligence: a brief guide for authors, reviewers, and readers-from the Radiology editorial board. Radiology 294(3):487–489 31891322 10.1148/radiol.2019192515

[CR37] Boulenger A, Luo Y, Zhang C, Zhao C, Gao Y, Xiao M, Zhu Q, Tang J (2023) Deep learning-based system for automatic prediction of triple-negative breast cancer from ultrasound images. Med Biol Eng Comput 61(2):567–578 36542320 10.1007/s11517-022-02728-4 PMC9852203

[CR23] Chen Z, Wang M, Feng R, Su M, Torres-de la Roche LA et al (2021) A machine learning model to predict the triple negative breast cancer immune subtype. Front Immunol 12:749459 34603338 10.3389/fimmu.2021.749459 PMC8484710

[CR48] Dodington DW, Lagree A, Tabbarah S, Mohebpour M, Sadeghi-Naini A, Tran WT et al (2021) Analysis of tumor nuclear features using artificial intelligence to predict response to neoadjuvant chemotherapy in high-risk breast cancer patients. Breast Cancer Res Treat 186:379–389 33486639 10.1007/s10549-020-06093-4

[CR19] Ensenyat-Mendez M, Llinàs-Arias P, Orozco JIJ, Íñiguez-Muñoz S, Salomon MP, Sesé B et al (2021) Current triple-negative breast cancer subtypes: dissecting the most aggressive form of breast cancer. Front Oncol 11:681476 34221999 10.3389/fonc.2021.681476 PMC8242253

[CR41] Gadag S, Sinha S, Nayak Y, Garg S, Nayak UY (2020) Combination therapy and nanoparticulate systems: smart approaches for the effective treatment of breast cancer. Pharmaceutics 12:524 32521684 10.3390/pharmaceutics12060524 PMC7355786

[CR44] Gautam P, Jaiswal A, Aittokallio T, Al-Ali H, Wennerberg K (2019) Phenotypic screening combined with machine learning for efficient identification of breast cancerselective therapeutic targets. Cell Chem Biol 26:970e4–9e4 31056464 10.1016/j.chembiol.2019.03.011 PMC6642004

[CR46] Guan H, Su Y, Guo W, Chen C, Xie X, Lv XA (2023) Prognostic model of genetic markers for triple-negative breast Cancer based on Machine Learning and Bioinformatics Analysis. Stud Health Technol Inf 308:303–312 10.3233/SHTI230854 38007754

[CR40] Hou X, Li X, Han Y, Xu H, Xie Y, Zhou T, Xue T, Qian X, Li J, Wang HC, Yan J, Guo X, Liu Y, Liu J (2024) Triple-negative breast cancer survival prediction using artificial intelligence through integrated analysis of tertiary lymphoid structures and tumor budding. Cancer 15:1499–1512 10.1002/cncr.35261 38422056

[CR14] Hu Z et al (2020) Multi-cancer analysis of clonality and the timing of systemic spread in paired primary tumors and metastases. Nat Genet 52:701–708 32424352 10.1038/s41588-020-0628-z PMC7343625

[CR20] Hu H, Tong K, Tsang JY, Ko CW, Tam F, Loong TC, Tse GM (2024) Subtyping of triple-negative breast cancers: its prognostication and implications in diagnosis of breast origin. ESMO Open 9:102993 38613910 10.1016/j.esmoop.2024.102993 PMC11024544

[CR30] Huang Y, Wei L, Hu Y, Shao N, Lin Y, He S et al (2021) Multi-parametric MRI-based radiomics models for predicting molecular subtype and androgen receptor expression in breast cancer. Front Oncol 11:706733 34490107 10.3389/fonc.2021.706733 PMC8416497

[CR47] Huang Z, Shao W, Han Z, Alkashash AM, Dela SC, Parwani AV, Nitta H, Hou Y, Wang T, Salama P, Rizkalla M, Zhang J, Huang K, Li Z (2023) Artificial intelligence reveals features associated with breast cancer neoadjuvant chemotherapy responses from multi-stain histopathologic images. NPJ Precis Oncol 27:14 10.1038/s41698-023-00352-5 PMC9883475 36707660

[CR49] Irajizad E, Wu R, Vykoukal J, Murage E, Spencer R, Dennison JB et al (2022) Application of artificial intelligence to plasma metabolomics profiles to predict response to neoadjuvant chemotherapy in triple-negative breast cancer. Front Artif Intell 5:876100 36034598 10.3389/frai.2022.876100 PMC9403735

[CR26] Jé zé quel P, Kerdraon O, Hondermarck H, Gué rin-Charbonnel C, Lasla H, Gouraud W et al (2019) Identification of three subtypes of triple-negative breast cancer with potential therapeutic implications. Breast Cancer Res 21:65–14 31101122 10.1186/s13058-019-1148-6 PMC6525459

[CR5] Jiang Y, Yang M, Wang S, Li X, Sun Y (2020) Emerging role of deep learning-based artifcial intelligence in tumor pathology. Cancer Commun (lond) 40(4):154–166 32277744 10.1002/cac2.12012 PMC7170661

[CR50] Kim J, Yu D, Kwon Y, Lee KS, Sim SH, Kong SY et al (2020) Genomic characteristics of triple-negative breast cancer nominate molecular subtypes that predict chemotherapy response. Mol Cancer Res 18:253–263 31704731 10.1158/1541-7786.MCR-19-0453

[CR45] Kothari C, Osseni MA, Agbo L, Ouellette G, Déraspe M, Laviolette F et al (2020) Machine learning analysis identifies genes differentiating triple negative breast cancers. Sci Rep 10:10464 32591639 10.1038/s41598-020-67525-1 PMC7320018

[CR7] Kumar Y, Koul A, Singla R, Ijaz MF (2022) Artifcial intelligence in disease diagnosis: a systematic literature review, synthesizing framework and future research agenda. J Ambient Intell Humaniz Comput 14:1–28 10.1007/s12652-021-03612-z PMC8754556 35039756

[CR29] Leithner D, Mayerhoefer ME, Martinez DF, Jochelson MS, Morris EA, Thakur SB et al (2020) Non-invasive assessment of breast cancer molecular subtypes with multiparametric magnetic resonance imaging radiomics. J Clin Med 9:1853 32545851 10.3390/jcm9061853 PMC7356091

[CR34] Li H, Ye J, Liu H, Wang Y, Shi B, Chen J et al (2021) Application of deep learning in the detection of breast lesions with four different breast densities. Cancer Med 10:4994–5000 34132495 10.1002/cam4.4042 PMC8290249

[CR38] Li S, Zhang N, Zhang H et al (2023) Artificial intelligence learning landscape of triple-negative breast cancer uncovers new opportunities for enhancing outcomes and immunotherapy responses. J Big Data 10:132

[CR22] Liu J, Su R, Zhang J, Wei L (2021) Classification and gene selection of triple-negative breast cancer subtype embedding gene connectivity matrix in deep neural network. Brief Bioinform 22(5):bbaa395 33415328 10.1093/bib/bbaa395

[CR35] Ma M, Liu R, Wen C, Xu W, Xu Z, Wang S et al (2022) Predicting the molecular subtype of breast cancer and identifying interpretable imaging features using machine learning algorithms. Eur Radiol 32:1652–1662 34647174 10.1007/s00330-021-08271-4

[CR2] Mendelson EB (2019) Artifcial intelligence in breast imaging: potentials and limitations. Am J Roentgenol 212(2):293–299 30422715 10.2214/AJR.18.20532

[CR15] Pandy JGP et al (2019) Triple negative breast cancer and platinum-based systemic treatment: a meta-analysis and systematic review. BMC Cancer 19:1065 31703646 10.1186/s12885-019-6253-5 PMC6839096

[CR10] Pelayo S, Bouaud J, Blancafort C, Lamy JB, Sekar BD, Larburu N et al (2021) Preliminary qualitative and quantitative evaluation of DESIREE, a decision support platform for the management of primary breast cancer patients. AMIA Annu Symp Proc 2020:1012–1021 33936477 PMC8075492

[CR56] Perez-Lopez R, Ghaffari Laleh N, Mahmood F et al (2024) A guide to artificial intelligence for cancer researchers. Nat Rev Cancer 24:427–441 38755439 10.1038/s41568-024-00694-7

[CR33] Porembka JH, Ma J, Le-Petross HT (2020) Breast density, MR imaging biomarkers, and breast cancer risk. Breast J 26:1535–1542 32654416 10.1111/tbj.13965

[CR9] Redjdal A, Bouaud J, Guézennec G, Gligorov J, Seroussi B (2021) Reusing decisions made with one decision support system to assess a second decision support system: introducing the notion of complex cases. Stud Health Technol Inf 281:649–653 10.3233/SHTI210251 34042656

[CR32] Romeo V, Kapetas P, Clauser P, Baltzer P, Rasul S, Gibbs P, Hacker M, Woitek R, Pinker K, Helbich TH (2022) A simultaneous multiparametric 18F-FDG PET/MRI Radiomics Model for the diagnosis of Triple negative breast Cancer. Cancers 14:3944 36010936 10.3390/cancers14163944 PMC9406327

[CR16] Saini KS, Punie K, Twelves C, de Bortini S, Anderson AE, Criscitiello S, Awada C, Loi A (2021) Antibody-drug conjugates, immune-checkpoint inhibitors, and their combination in breast cancer therapeutics. Expert Opin Biol Ther 21:945–962 34043927 10.1080/14712598.2021.1936494

[CR28] Shamir SB, Sasson AL, Margolies LR, Mendelson DS (2024) New frontiers in breast Cancer imaging: the rise of AI. Bioeng (Basel) 11:451 10.3390/bioengineering11050451 PMC11117903 38790318

[CR43] Thakur V, Kutty RV (2019) Recent advances in nanotheranostics for triple negative breast cancer treatment. J Exp Clin Cancer Res 38:430 31661003 10.1186/s13046-019-1443-1 PMC6819447

[CR18] Tsang JY, Tse GM (2023) Update on triple-negative breast cancers - highlighting subtyping update and treatment implication. Histopathology 82:17–35 36468263 10.1111/his.14784

[CR51] Tsopra R, Fernandez X, Luchinat C, Alberghina L, Lehrach H, Vanoni M et al (2021) A framework for validating AI in precision medicine: considerations from the European ITFoC consortium. BMC Med Inf Decis Mak 21:274 10.1186/s12911-021-01634-3 PMC8487519 34600518

[CR42] Tsou LK, Yeh SH, Ueng SH, Chang CP, Song JS, Wu MH et al (2020) Comparative study between deep learning and QSAR classifications for TNBC inhibitors and novel GPCR agonist discovery. Sci Rep 10:16771 33033310 10.1038/s41598-020-73681-1 PMC7545175

[CR6] Ullah R, Khan S, Ishtiaq I, Shahzad S, Ali H, Bilal (2020) M.Cost efective and efcient screening of Alzheimer disease with Raman spectroscopy and machine learning algorithms. Photodiagn Photodyn Ther 32:101963 10.1016/j.pdpdt.2020.101963 33321570

[CR11] Vagia E, Mahalingam D, Cristofanilli M (2020) The landscape of targeted therapies in TNBC. Cancers 12:916 32276534 10.3390/cancers12040916 PMC7226210

[CR8] Valieris R, Amaro L, Osório T, Bueno AP, Rosales Mitrowsky RA, Carraro DM, Nunes DN, Dias-Neto E, Silva ITD (2020) Deep learning predicts underlying features on pathology images with therapeutic relevance for breast and gastric cancer. Cancers (Basel) 12(12):3687 33316873 10.3390/cancers12123687 PMC7763049

[CR24] Wang M, Feng R, Chen Z, Shi W, Li C, Liu H et al (2022) Identification of cancer-associated fibroblast subtype of triple-negative breast cancer. J Oncol 2022:6452636 35505821 10.1155/2022/6452636 PMC9057104

[CR53] Wang X, Du Y, Yang S, Zhang J, Wang M, Zhang J, Yang W, Huang J, Han X (2023) RetCCL: clustering-guided contrastive learning for whole-slide image retrieval. Med Image Anal 83:102645 36270093 10.1016/j.media.2022.102645

[CR36] Wu T, Sultan LR, Tian J, Cary TW, Sehgal CM (2019) Machine learning for diagnostic ultra sound of triple-negative breast cancer. Breast Cancer Res Treat 173:365–373 30343454 10.1007/s10549-018-4984-7

[CR3] Yan S, Li J, Wu W (2023) Artificial intelligence in breast cancer: application and future perspectives. J Cancer Res Clin Oncol 149(17):16179–16190 37656245 10.1007/s00432-023-05337-2 PMC11797688

[CR13] Yu T, Di G (2017) Role of tumor microenvironment in triple-negative breast cancer and its prognostic signifcance. Chin J Cancer Res 29:237–252 28729775 10.21147/j.issn.1000-9604.2017.03.10 PMC5497211

[CR21] Yu X, Liu Y, Chen M (2022) Reassessment of reliability and reproducibility for triplenegative breast cancer subtyping. Cancers 14:2571 35681552 10.3390/cancers14112571 PMC9179838

[CR12] Zhang X, Yeung KT (2023) Metastatic triple-negative breast Cancer. Curr Breast Cancer Rep 15:288–297

[CR17] Zhao S, Zuo WJ, Shao ZM, Jiang YZ (2020) Molecular subtypes and precision treatment of triple-negative breast cancer. Ann Transl Med 8:499 32395543 10.21037/atm.2020.03.194 PMC7210152

[CR31] Zhou Z, Adrada BE, Candelaria RP, Elshafeey NA, Boge M, Mohamed RM, Pashapoor S, Sun J, Xu Z et al (2023) Prediction of pathologic complete response to neoadjuvant systemic therapy in triple negative breast cancer using deep learning on multiparametric MRI. Sci Rep 13(1):1171 36670144 10.1038/s41598-023-27518-2 PMC9859781

